# Electrocardiographic Frontal QRS-T Angle Is Independently Associated with the Presence of Celiac Disease and Disease Duration

**DOI:** 10.3390/diagnostics15020187

**Published:** 2025-01-15

**Authors:** Betül Ayça Yamak, İbrahim Ethem Güven, Mustafa Candemir

**Affiliations:** 1 Department of Cardiology, Hopa State Hospital, 08600 Artvin, Turkey; 2 Department of Gastroenterology, Yenimahalle Education and Research Hospital, 06370 Ankara, Turkey; drethemg@gmail.com; 3 Department of Cardiology, Faculty of Medicine, Gazi University, 06560 Ankara, Turkey; mstfcndmr@hotmail.com

**Keywords:** celiac disease, electrocardiography, frontal QRS-T angle

## Abstract

**Background:** The impact of Celiac Disease (CD) is not only limited to the intestinal system, but extraintestinal manifestations may also be seen. In this context, cardiac manifestations have recently been the focus of attention. This study aimed to evaluate myocardial repolarization properties in CD patients by assessing the frontal QRS-T Angle (fQRS-T) on electrocardiography (ECG). **Methods:** A total of 302 patients, including 150 CD patients and 152 control group patients, were included in the study. ECG parameters, including fQRS-T, QRS interval, and QTc interval, were calculated for each patient and compared between the groups. In addition, the relationship of these ECG parameters with disease duration was also analyzed. **Results:** The median disease duration was 38.5 (16 to 96) months in the CD group. Significantly wider QRS interval (92 (86 to 96) vs. 83 (76.3 to 93), *p* < 0.001) and fQRS-T (23 (13 to 37) vs. 18 (6.3 to 27), *p* < 0.001) values were observed in the CD group. Among CD patients, those with longer disease duration (>38.5 months) exhibited significantly wider QRS intervals (94 (88 to 98) vs. 88 (84 to 94), *p* < 0.001) and frontal QRS-T angles (29 (14 to 47) vs. 16 (10 to 25), *p* < 0.001) compared to those with shorter disease duration. A positive correlation between the disease duration and fQRS-T was also demonstrated (r = 0.478, *p* < 0.001). Multivariable logistic regression identified QRS interval (OR: 1.060, 95% CI: 1.032–1.088, *p* < 0.001) and frontal QRS-T angle (OR: 1.028, 95% CI: 1.013–1.043, *p* < 0.001) as independent predictors of CD. Additionally, the QRS interval (OR: 1.066, 95% CI: 1.012–1.124, *p* = 0.016) and frontal QRS-T angle (OR: 1.021, 95% CI: 1.003–1.038, *p* = 0.021) were significant predictors of longer disease duration. A linear regression analysis confirmed that disease duration was a stronger predictor of frontal QRS-T angle widening (B: 0.389, 95% CI: 0.102–0.677, *p* < 0.001) compared to age (B: 0.184, 95% CI: 0.123–0.245, *p* = 0.008). **Conclusions:** In this study, we demonstrated that chronic inflammation secondary to CD may have negative effects on cardiac repolarization and that this effect is closely related to disease duration.

## 1. Introduction

Celiac disease (CD) is an autoimmune reaction triggered by the consumption of gluten, a protein in wheat, rye, and barley [1]. Beyond its well-documented gastrointestinal effects, CD has been increasingly recognized as a systemic disease with significant implications for the neurological, endocrine, and cardiovascular systems [2]. The cardiovascular effects of CD, although not as well studied as the gastrointestinal and neurological effects, are beginning to receive attention because of their potential clinical importance. Chronic systemic inflammation, a defining characteristic of autoimmune disorders, has been linked to the onset and progression of cardiovascular diseases such as atherosclerosis, arrhythmias, and myocardial dysfunction [3,4]. Chronic inflammation in CD may contribute to endothelial dysfunction, oxidative stress, and altered vascular reactivity, thereby increasing the risk of cardiovascular diseases. These mechanisms are consistent with findings in other autoimmune conditions, where systemic inflammation is a key driver of cardiovascular pathology [5].

Electrocardiography (ECG) has an essential place in cardiovascular risk assessment. Various parameters obtained from the ECG, such as the QT interval, T-wave axis, and QRS-T angle, have been studied as markers of cardiovascular health [6,7,8]. Among these, the frontal QRS-T angle has emerged as a significant predictor of adverse cardiac events. The QRS-T angle is a significant parameter that reflects the spatial difference between ventricular depolarization (QRS axis) and repolarization (T-wave axis). An increased QRS-T angle has been associated with a higher risk of adverse cardiac events, including arrhythmias, myocardial infarction, and sudden cardiac death [9,10]. In the context of CD, systemic inflammation may influence cardiac electrophysiology, leading to changes in ECG parameters [11]. Specifically, the widening of the QRS-T angle in CD patients could reflect the impact of chronic inflammation on ventricular depolarization and repolarization processes. Previous studies have suggested that inflammation-related changes in cardiac electrical activity may serve as early indicators of subclinical cardiovascular dysfunction [12]. The main purpose of this study is to examine the frontal QRS-T angle in celiac patients and to evaluate the relationship between this angle and disease duration. In addition, our findings aim to determine a potential parameter that can be used in cardiovascular risk assessment in celiac patients by illuminating the effects of chronic inflammation on the cardiovascular system.

## 2. Materials and Methods

### 2.1. Study Populations

This retrospective observational study was carried out in the gastroenterology clinic of the Yenimahalle Education and Research Hospital. Patients who were followed in the outpatient clinic of our gastroenterology department with a diagnosis of CD between January 2021 and May 2024 were evaluated for inclusion in this study. The diagnosis of CD was based on the clinical symptoms, endoscopic examination, and celiac serology combined with histopathological evaluation of intestinal biopsy.

**Figure 1:**
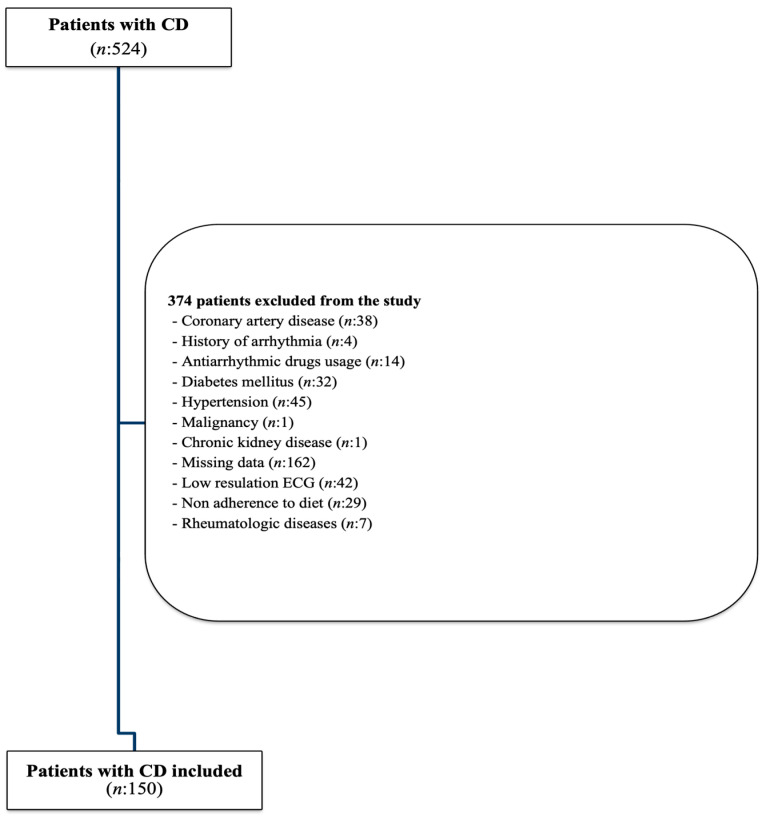
Flowchart of patient inclusion.

All participants were analyzed for eligibility for inclusion in the study using the following exclusion criteria established for the study design: (i) patients with missing data; (ii) history of coronary artery disease (previously diagnosed by anamnesis in outpatient clinic visits or identified with The International Classification of Diseases codes); (iii) ECG findings showing ischemic manifestations; (iv) those with left ventricular dysfunction and/or severe valvular disease detected on echocardiographic evaluation; (v) any cancer; (vi) chronic diseases including diabetes mellitus, autoimmune disorders and rheumatologic diseases, respiratory diseases, liver and kidney failure, hypo/hyperthyroidism, or arterial hypertension; (vii) electrolyte abnormalities in blood tests; (viii) history of arrhythmia, bundle branch block patterns, or intraventricular conduction delay; (ix) patients with a history of interventional or surgical treatment of arrhythmia and/or receiving any antiarrhythmic drug treatment; (x) patients under 18 years old; (xi) patients with a history of other comorbidities or medications that may affect the ECG parameters. In addition, patients with low-resolution ECGs and insufficient ECG signal quality were also excluded from the study since the calculations to be performed on ECG require precise measurement, and ECG resolution and quality are crucial in this concept. Lastly, patients with a diagnosis of CD of less than 12 months and patients who had not adhered to a gluten-free diet for the last 6 months were not included in the study. The flowchart illustrates the patient inclusion and exclusion process (Figure 1).

The age- and gender-matched control groups were recruited prospectively from consecutive patients who were admitted to the outpatient clinic of our gastroenterology department with dyspepsia complaints, and no underlying pathology was demonstrated in either anamnesis, laboratory, or endoscopic evaluations. In addition, each patient in the control group was also analyzed for compliance with the exclusion criteria mentioned above.

Ethical approval was obtained from the Ankara Training and Research Hospital Scientific Research Assessment and Ethics Committee (Approval No: E-24-293).

### 2.2. Data Collection and Electrocardiographic Evaluation

The electronic hospital data system was used to obtain data on demographic features such as age, gender, diagnosis date, and body mass index (BMI). Anamnesis data such as comorbidities, medication history, history of previous surgery, and smoking status were obtained from outpatient clinic assessment notes and patient evaluation forms. Detailed blood test results, including complete blood count, lipid profile, and biochemical data, were extracted from hospital digital records.

Standard 12-lead ECGs were obtained from all participants using the Nihon Kohden ECG device (Model-2350, Nishiochiai, Shinjuku-ku, Tokyo, Japan) and thoroughly reviewed to ensure accurate measurements and data quality for the study. ECG recordings were performed at a paper speed of 25 mm/s and a voltage calibration of 10 mm/mV, with electrodes placed in standard anatomical positions following a 10-min rest period in the supine position. To achieve objective and sensitive measurements, the QRS and T axis values were retrieved from the automated measurement outputs on the printed version of the ECGs. The frontal QRS-T angle was calculated as the absolute difference between the QRS axis and T axis (frontal QRS-T angle =│QRS axis − T axis│), derived from the limb leads on a standard 12-lead ECG. If the measured angle was greater than 180°, it had to be subtracted from 360°. All calculations were performed by a Cardiologist blinded to the subjects’ data in the assessment phase.

### 2.3. Statistical Analysis

The statistical analyses were performed using the SPSS 25.0 (SPSS Inc., Chicago, IL, USA) software for Windows. The normality distribution of numerical variables was evaluated by using the Kolmogorov-Smirnov test. Normally distributed variables were presented as mean ± SD, and abnormally distributed variables were presented as median (interquartile range) values. When comparing continuous variables for more than two groups, a one-way analysis of variance or the Kruskal–Wallis test was used, whichever was appropriate. Bonferroni correction was applied for post-hoc tests. Categorical variables were presented as numbers and percentages. The chi-square test was used to compare these variables between the groups. Univariable and multivariable logistic regression analyses were performed to determine the independent predictors of celiac disease. Variables with *p* < 0.25 in univariable logistic regression and believed to be of clinical importance were included in a multivariable logistic regression model. Linear regression analysis was performed to evaluate the effect of disease duration and age on QRS-T angle widening. A two-sided *p* < 0.05 was considered significant.

## 3. Results

The study population consisted of 302 individuals, 150 in the CD group and 152 in the control group. The median age in the CD group was 39 (26 to 49) years, while 101 individuals (67%) were female. In addition, the median QRS interval was 92 (86 to 96) ms, QTc interval 423 (406.8 to 438) ms, frontal QRS-T angle 23 (13 to 37) (°), and disease duration 38.5 (16 to 96) months in patients with CD (Table 1).

Age (OR: 0.973, 95% CI: 0.954–0.993, *p* = 0.008), QRS interval (OR: 1.060, 95% CI: 1.032–1.088, *p* < 0.001), and frontal QRS-T angle (OR: 1.028, 95% CI: 1.013–1.043, *p* < 0.001) were found to be independent predictors for CD in the multivariable regression analysis on the study group (Table 2).

The study population was divided into three groups (control group = group 1, patients with disease duration <38.5 months = group 2, patients with disease duration >38.5 months = group 3). There was no statistically significant difference in demographic characteristics such as gender, smoking status, and BMI among the three groups (*p* > 0.05 for all parameters). Also, there was no statistically significant difference in laboratory findings (glucose, creatinine, sodium, potassium, calcium, sedimentation, CRP) between the three groups (*p* > 0.05 for all parameters). QRS interval (group 1 = 83 (76.3 to 93) ms, group 2 = 88 (84 to 94) ms, group 3 = 94 (88 to 98) ms, *p* < 0.001) and frontal QRS-T angle (group 1 = 18 (6.3 to 27), group 2 = 16 (10 to 25), group 3 = 29 (14 to 47), *p* < 0.001) values were wider in the group with longer disease duration (Table 3).

QRS interval (OR: 1.066, 95% CI: 1.012–1.124, *p* = 0.016) and frontal QRS-T angle (OR: 1.021, 95% CI: 1.003–1.038, *p* = 0.021) were found to be independent predictors for long disease duration in the multivariable regression analysis (Table 4).

A linear regression analysis was performed to evaluate the effects of disease duration and age on QRS-T angle widening. It was observed that disease duration was greater than the effect of age. (B: 0.184, 95% CI: 0.123–0.245, *p*: 0.008 for age; B: 0.389, 95% CI: 0.102–0.677, *p* < 0.001 for disease duration) (Table 5).

**Figure 2:**
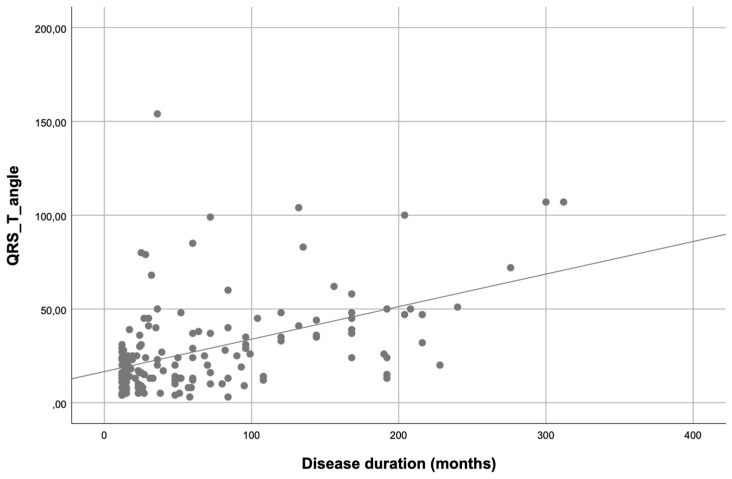
Scatter plot showing a positive linear correlation between frontal QRS-T angle and disease duration (R^2^ linear = 0.229; r = 0.478; *p* < 0.001).

Additionally, there was a moderate positive correlation between disease duration and frontal QRS-T angle value (r = 0.478, *p* < 0.001) (Figure 2).

## 4. Discussion

In this study, celiac patients demonstrated a significantly wider frontal QRS-T angle than the control group. Additionally, this widening became more pronounced with a longer duration of the disease.

**Figure 3:**
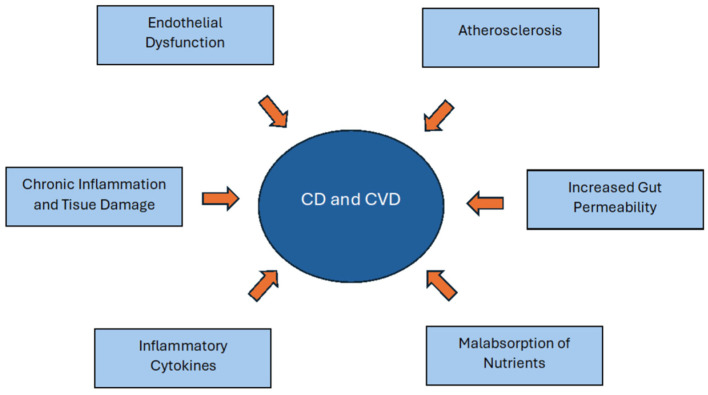
Factors associated with the development of cardiovascular diseases in patients with celiac disease.

In CD, sensitivity to gluten initiates an immune response that stimulates the production of specific antibodies, particularly tissue transglutaminase antibodies [13]. This immune activation leads to chronic inflammation that is not confined to the gastrointestinal tract but extends to other systemic manifestations. Chronic inflammation in CD can affect multiple organ systems, including the cardiovascular, neurological, and endocrine systems [2,14]. Autoimmune diseases, including CD, have been strongly associated with an increased risk of cardiovascular diseases such as atherosclerosis, myocardial dysfunction, and arrhythmias [11]. This relationship is largely attributed to the pro-inflammatory state observed in these conditions [15]. Pro-inflammatory cytokines, including interleukin-6 (IL-6), tumor necrosis factor-alpha (TNF-α), and CRP, are often elevated in CD and play a pivotal role in systemic inflammation, contributing not only to vascular inflammation and the destabilization of atherosclerotic plaques but also to the pathophysiology of a wide spectrum of cardiovascular diseases [16,17]. Figure 3 illustrates the potential mechanisms linking CD to cardiovascular complications.

Emerging evidence has identified an increased prevalence of atrial fibrillation in patients with CD. For instance, a large population-based study reported a significantly higher risk of atrial fibrillation among individuals with CD compared to controls, highlighting the potential impact of chronic inflammation on cardiac electrophysiology [18,19]. Additionally, untreated CD has been linked to prolonged QTc intervals, a well-established marker of ventricular repolarization abnormalities associated with an increased risk of ventricular arrhythmias and sudden cardiac death [20]. These findings suggest that chronic inflammation and nutritional deficiencies in CD may have deleterious effects on myocardial function and electrical conduction.

The QRS-T angle, a measurement derived from vectorcardiography (VCG) analysis, has been widely researched since 1934 when Wilson and colleagues introduced the concept of the “ventricular gradient”, defined as the vectorial sum of the spatial QRS-T angle [21]. The frontal QRS-T angle provides a practical approach for assessing cardiac electrical activity, serving as a readily obtainable parameter in a two-dimensional plane using a standard 12-lead ECG. Numerous studies have investigated the prognostic significance of a wide QRS-T angle across different populations, highlighting its association with adverse cardiovascular outcomes [22,23,24]. Aro et al. showed that a wide QRS-T angle increased the risk of total mortality in the general population with a 30-year follow-up [9]. A wide QRS-T angle is a powerful and independent predictor of fatal arrhythmias. The increased risk linked to a wide QRS-T angle is primarily attributed to an abnormal T-wave axis, which may act as an early indicator of myocardial damage [9]. Differences in the QRS-T angle have been noted among patients with normal ventricular conduction and those with different pacemaker implantation techniques [25].

In the study conducted by Whang et al., it was found that a wide QRS-T angle increased the risk of mortality in men in individuals without cardiovascular disease. However, no significant association was observed in women, suggesting that gender may affect the prognostic value [26]. Lown et al. found that a wide QRS-T angle was associated with total mortality in patients with acute coronary syndrome, while Gotsman et al. showed that a wide QRS-T angle increased the risk of mortality in both sexes in individuals with chronic heart failure [27,28]. Additionally, it has been observed that inflammation and cardiac wall stress/injury markers are higher in individuals with wide QRS-T angles [29].

The findings in our study confirm this result in CD, which is known to be a chronic inflammatory process. The widening of the QRS-T angle, especially as disease duration increases, suggests that inflammation may have a cumulative effect on the cardiovascular system in the long term. This situation reveals that chronic inflammation may increase cardiovascular risk factors over time by disrupting cardiac electrophysiology. The role of the QRS-T angle as a cardiovascular risk indicator in celiac patients has not been investigated in the literature.

The QRS-T angle has not been previously studied as a cardiovascular risk marker in celiac patients, making this research a novel addition to the field. By exploring the relationship between the QRS-T angle and CD, this study adds valuable insights into how this parameter could be used to assess cardiovascular impacts in this patient population. These findings underscore the need for further research involving larger, more diverse populations to confirm and expand upon these results. Multicenter, randomized controlled trials would provide stronger evidence and help establish the clinical utility of the QRS-T angle in this context. Moreover, future studies incorporating carefully controlled medication regimens and thorough assessments of comorbidities would offer a more comprehensive understanding of the cardiovascular effects of CD. Such investigations could contribute to a better representation of the general population and enhance knowledge of how chronic inflammatory diseases like celiac disease influence cardiac health.

This study has some limitations, including its retrospective design, small number of patients, and single-center nature. This study does not include follow-up data to assess the association between the QRS-T angle and specific cardiovascular outcomes, such as arrhythmias, myocardial infarction, or sudden cardiac death.

## 5. Conclusions

Our study suggests chronic inflammation in celiac disease may lead to widening the QRS-T angle. Furthermore, a wider QRS-T angle is observed with increasing disease duration, suggesting the cumulative effect of long-term inflammatory burden on the cardiovascular system.

## Figures and Tables

**Table 1:** Baseline characteristics, laboratory, and 12-lead electrocardiographic parameters in patients with celiac disease.

	Celiac (*n* = 150)
Age, years	39 (26–49)
Sex (Female), *n* (%)	101 (67)
Smoking, *n* (%)	45 (30)
BMI, kg/m^2^	25.1 (24–26.3)
Glucose, mg/dL	93 (84–110)
WBC, ×10^3^/mL	7 ± 1.6
Hemoglobin, g/dL	13.6 ± 1.6
AST, U/L	18 (15–23.3)
ALT, U/L	19.5 (16–25)
Creatinine, mg/dL	0.7 (0.6–0.9)
Sodium, mmol/L	140 ± 2.3
Potassium, mmol/L	4.3 (4.1–4.5)
Calcium, mg/dL	9.5 ± 0.5
Sedimentation rate, mm/h	8 (5–12)
CRP, mg/dL	1 (0.5–2.9)
Heart rate, beat/min	78 (69–87.3)
QRS interval, ms	92 (86–96)
QTc interval, ms	423 (406.8–438)
Frontal QRS-T angle, (°)	23 (13–37)
Disease duration, months	38.5 (16–96)

Results are expressed as mean ± SD, median (IQR), or frequency (%). SD: Standard deviation, IQR: Interquartile range, BMI: body mass index, WBC: white blood cell, AST: aspartate aminotransferase, ALT: alanine aminotransferase, CRP: C-reactive protein, QTc: Corrected QT interval. Statistically significant results (*p* < 0.05) are shown in bold type.

**Table 2:** Multivariable logistic regression analyses of predictors for celiac disease.

	Univariate Analysis				Multivariable Analysis			
		95% CI				95% CI		
	OR	Lower	Upper	*P*	OR	Lower	Upper	*P*
Age	0.990	0.973	1.007	0.226	0.973	0.954	0.993	**0.008**
Sex	1.308	0.816	2.097	0.265	1.374	0.812	2.325	0.236
Smoking	1.122	0.682	1.848	0.650				
BMI	0.996	0.918	1.081	0.924				
Sedimentation rate	0.982	0.950	1.015	0.277				
CRP	0.930	0.850	1.017	0.113	0.936	0.848	1.033	0.190
Heart rate	0.994	0.977	1.011	0.486				
QRS interval	1.065	1.039	1.091	<0.001	1.060	1.032	1.088	**<0.001**
QTc interval	0.997	0.993	1.001	0.107	0.996	0.992	1.001	0.139
Frontal QRS-T angle	1.030	1.015	1.045	<0.001	1.028	1.013	1.043	**<0.001**

BMI: body mass index, CRP: C-reactive protein, QTc: Corrected QT interval. Statistically significant results (*p* < 0.05) are shown in bold type.

**Table 3:** Baseline characteristics. Laboratory and 12-lead electrocardiographic parameters of the median celiac disease duration.

	Control (*n* = 152)	Disease Duration, <38.5 Months (*n* = 75)	Disease Duration, >38.5 Months (*n* = 75)	*P*
Age, years	41 (31–51)	33 (24–47) ^a^	43 (32–51)	**0.009**
Sex (Female), *n* (%)	93 (61)	46 (61)	55 (73)	0.166
Smoking, *n* (%)	42 (28)	21 (28)	24 (32)	0.779
BMI, kg/m^2^	25 (23–26.7)	25.3 (24.5)	25 (23.3–26.3)	0.724
Glucose, mg/dL	95.5 (86.3–107)	93 (84–110)	93 (86–108)	0.774
WBC, ×10^3^/mL	7.1 ± 2.1	6.8 ± 1.6	7 ± 1.6	0.540
Hemoglobin, g/dL	13.6 ± 1.9	13.7 ± 1.7	13.5 ± 1.4	0.671
AST, U/L	20 (14–29.3)	18 (16–24)	17 (14–23)	0.110
ALT, U/L	20 (16–25)	18 (16–25)	20 (15–25)	0.427
Creatinine, mg/dL	0.7 (0.6–0.9)	0.7 (0.6–0.9)	0.7 (0.6–0.8)	0.609
Sodium, mmol/L	140 ± 2.9	140 ± 2.3	140 ± 2.4	0.639
Potassium, mmol/L	4.2 (3.9–4.5)	4.3 (4.1–4.5)	4.3 (4.1–4.5)	0.190
Calcium, mg/dL	9.6 ± 0.7	9.5 ± 0.5	9.5 ± 0.4	0.807
Sedimentation rate, mm/h	7.5 (4–11)	7 (5–10)	8 (5–13)	0.235
CRP, mg/dL	1.2 (0.5–3.4)	1.1 (0.5–3)	1 (0.5–2.5)	0.265
Heart rate, beat/min	78.5 (70.3–88)	75.7 ± 12.8	76.6 ± 13.2	0.099
QRS interval, ms	83 (76.3–93) ^a^	88 (84–94) ^b^	94 (88–98) ^c^	**<0.001**
QTc interval, ms	425 (394–461.1)	423.6 ± 24.2	422.7 ± 23.4	0.259
Frontal QRS-T angle, (°)	18 (6.3–27) ^a^	16 (10–25) ^a^	29 (14–47) ^b^	**<0.001**

Results are expressed as mean ± SD, median (IQR), or frequency (%). ^a,b,c^ Denotes statistically significant cells after Bonferroni adjustment. SD: Standard deviation, IQR: Interquartile range, BMI: body mass index, WBC: white blood cell, AST: aspartate aminotransferase, ALT: alanine aminotransferase, CRP: C-reactive protein, QTc: Corrected QT interval. Statistically significant results (*p* < 0.05) are shown in bold type.

**Table 4:** Multivariable logistic regression analyses of predictors for median celiac disease duration.

	Univariate Analysis				Multivariable Analysis			
		95% CI				95% CI		
	OR	Lower	Upper	*P*	OR	Lower	Upper	*P*
Age	1.034	1.009	1.060	0.008	1.020	0.993	1.048	0.149
Sex	1.734	0.868	3.461	0.119	1.514	0.707	3.240	0.286
Smoking	0.826	0.411	1.663	0.593				
BMI	0.890	0.740	1.069	0.213	0.845	0.691	1.034	0.102
Sedimentation rate	1.058	0.993	1.127	0.083	1.054	0.985	1.128	0.129
CRP	1.022	0.894	1.164	0.753				
Heart rate	1.006	0.981	1.031	0.654				
QRS interval	1.084	1.034	1.136	0.001	1.066	1.012	1.124	**0.016**
QTc interval	0.998	0.985	1.012	0.817				
Frontal QRS-T angle	1.025	1.008	1.043	0.004	1.021	1.003	1.038	**0.021**

BMI: body mass index, CRP: C-reactive protein. Statistically significant results (*p* < 0.05) are shown in bold type.

**Table 5:** Relationship between frontal QRS-T angle and other parameters in celiac patients.

Variables	Multivariable Linear Regression Analysis							
	Model 1				Model 2			
	B	%95 CI		P	B	%95 CI		P
		Lower	Upper			Lower	Upper	
Age	0.184	0.123	0.245	0.008	-	-	-	-
Sex	−1.821	−10.482	6.840	0.678	−4.221	−12.223	3.782	0.299
BMI	1.257	−0.919	3.433	0.256	1.575	−0.426	3.575	0.122
CRP	0.179	−1.458	1.816	0.829	0.051	−1.445	1.546	0.947
QRS interval	0.582	0.067	1.097	0.027	−0.073	−0.607	0.461	0.787
QTc interval	0.087	−0.085	0.259	0.317	0.115	−0.043	0.273	0.152
Disease duration	-	-	-	-	0.389	0.102	0.677	<0.001

BMI: body mass index, CRP: C-reactive protein, QTc: Corrected QT interval.

## Data Availability

Dataset available on request from the authors.
